# An event-oriented diffusion-refinement method for sparse events completion

**DOI:** 10.1038/s41598-024-57333-2

**Published:** 2024-03-21

**Authors:** Bo Zhang, Yuqi Han, Jinli Suo, Qionghai Dai

**Affiliations:** 1 Department of Automation, Tsinghua University, Beijing, 100084 China; 2 Institute for Brain and Cognitive Sciences, Tsinghua University, Beijing, 100084 China; 3 Shanghai Artificial Intelligence Laboratory, Shanghai, 200232 China

**Keywords:** Computer science, Imaging and sensing

## Abstract

Event cameras or dynamic vision sensors (DVS) record asynchronous response to brightness changes instead of conventional intensity frames, and feature ultra-high sensitivity at low bandwidth. The new mechanism demonstrates great advantages in challenging scenarios with fast motion and large dynamic range. However, the recorded events might be highly sparse due to either limited hardware bandwidth or extreme photon starvation in harsh environments. To unlock the full potential of event cameras, we propose an inventive event sequence completion approach conforming to the unique characteristics of event data in both the processing stage and the output form. Specifically, we treat event streams as 3D event clouds in the spatiotemporal domain, develop a diffusion-based generative model to generate dense clouds in a coarse-to-fine manner, and recover exact timestamps to maintain the temporal resolution of raw data successfully. To validate the effectiveness of our method comprehensively, we perform extensive experiments on three widely used public datasets with different spatial resolutions, and additionally collect a novel event dataset covering diverse scenarios with highly dynamic motions and under harsh illumination. Besides generating high-quality dense events, our method can benefit downstream applications such as object classification and intensity frame reconstruction.

## Introduction

As a novel bio-inspired sensor, event cameras work in a different way from conventional intensity cameras via sensing asynchronous pixel-wise brightness changes. The working principle renders the sensor unique characteristics such as high sensitivity, low latency and high temporal resolution, which provide reliable visual information and wide applications in extreme environments, e.g. fast avoidance^1^, low-light/high dynamic range perception^2^ and high-speed imaging^3,4^ etc. However, such features are traded with spatial and temporal sparsity in the data stream. Firstly, only brightness changes exceeding a threshold can be recorded, and the outputs mostly locate salient moving edges and patterns. Although this issue can be alleviated by raising the sensitivity level but would bring more noise, imposing great challenges to the successive processing and analysis. Secondly, the readout speed may be limited by the hardware’s bandwidth (e.g. drone, PC etc.) even if the camera itself works in the full-capacity mode and causes missing entries in the data stream, which is especially severe in high-resolution and busy scenes. The above issues would degenerate or even fail many off-the-shelf event analysis algorithms working well on event streams in ordinary scenarios. To fully utilize the advantages of event cameras in challenging cases (e.g. low-light, high-speed), recovering the missing signals from the sparsely recorded event streams is of crucial importance, but remains an under-explored area.

**Figure 1:**
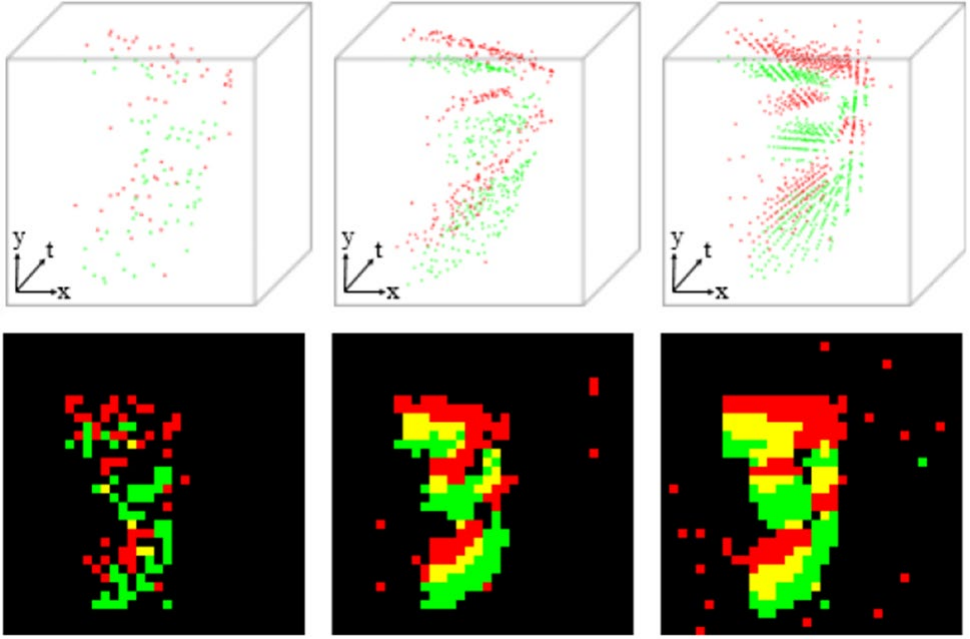
An exemplar demonstration of our event completion performance, in terms of 3D spatiotemporal cloud (upper) and accumulated 2D image (lower). left: the sub-sampled sparse sequence consisting of 128 events; middle: the completed counterpart; right: the ground truth. Green and red indicate positive and negative events respectively.

In analog to other event quality enhancement tasks, such as super-resolution^5–8^, joint denoising and super-resolution^8^^5^, one can convert raw events into 2D grid-based representation for algorithm development. This intuitive solution facilitates adapting the algorithms working on conventional image/video frames, but faces limitations in multiple aspects: assigning no or random timestamps to the output events would lose the temporal ordering information; the output event frames are non-binary, which deviates from the format of event data; the grid-based representation includes large proportion of event-free elements and thus the successive algorithms are storage demanding; such mismatch between the representation and the intrinsic structure might further lead to artificial results in recovered event streams and even harm the downstream analysis. In comparison, Li et al.^7^ proposed an inspiring strategy to super-resolve the events while maintaining the temporal information. However, the temporal precision is limited to milliseconds when using spiking neural network (SNN) for simulation, and far insufficient for microsecond responses of event cameras. An efficient algorithm making extensive use of the unique structure of event sequence and conforming to its format is highly demanded.

Event sequence can be formulated as a binary 3D data given a time duration (shown in the left of Fig. 1), with a four-element tuple (*x*, *y*, *t*, *p*) denoting the location, time instant and polarity of each event. In other words, the event occurrences compose a cloud, similar to the point cloud in 3D vision. Built on this representation, we propose an event data completion method based on the powerful generative discrete diffusion probabilistic model (DDPM), and develop an event-oriented deep network as the cornerstone. Our method works in a coarse-to-fine manner that firstly predicts a coarse distribution on the condition of sparse event sequences and then refines the generated events with the conditional input with a second sub-network. The final output of the network is a completed set of 3D events that can be transformed back to sequential format without losing temporal ordering information (middle column, Fig. 1). To validate our effectiveness in diverse scenarios, we collect a new dataset consisting of diverse challenging scenes and conduct experiments on it together with three public datasets with different spatial resolutions. Furthermore, we also show that our method can benefit downstream applications, including object classification and frame reconstruction.

In sum, this paper contributes in the following aspects:

- We propose to process a raw event sequence as an event cloud and recover the dense event signals underlying the recorded sparse event streams via a diffusion-based generative model.
- We develop an event-oriented network as the cornerstone of the diffusion model, which outputs complete dense events with better visual quality while maintaining temporal ordering information.
- We validate the advantageous performance of our approach and its wide applicability to diverse scenes on three public datasets with different resolutions, and show our superior performance on real challenging cases with a self-captured dataset.
- We conduct two downstream tasks using the completed events, i.e. object classification and intensity frame reconstruction, and obtain satisfying results, demonstrating the wide applications of our method.

## Problem statement

### Event formation

Let $I({\varvec{q}}_k,t_k)$ denote the brightness at pixel ${\varvec{q}}_k=(x_k,y_k)^T$ and time $t_k$. As an asynchronous sensor, an event camera senses pixel-wise illuminance changes, with each pixel independently responding to the change in the logarithmic brightness $L({\varvec{q}}_k,t_k)=\log (I({\varvec{q}}_k,t_k))$. Specifically, an event occurs when the brightness change since the last event at this location reaches a threshold $\pm C (C>0)$

1 $$\begin{aligned} |\textstyle L({\varvec{q}}_k,t_k)-L({\varvec{q}}_k,t_k-\Delta t_k)|\ge C, \end{aligned}$$

where $\Delta t_k$ is the time since the last event at ${\varvec{q}}$. An event sequence can be represented as a set of four-element tuples $\varepsilon (t_N)=\lbrace e_k\rbrace _{k=1}^N=\lbrace (t_k,x_k,y_k,p_k)\rbrace _{k=1}^N$ with microsecond resolution where $p_k$ is a binary value (1 or − 1) indicating the sign of the change in brightness.

**Figure 2:**
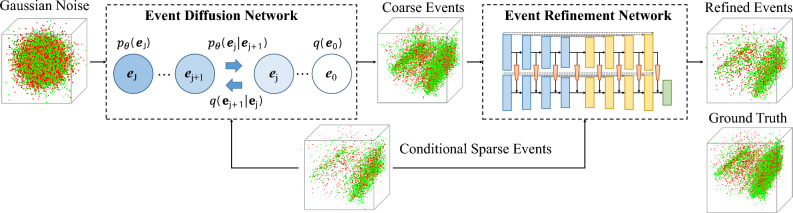
The overview of diffusion-based coarse-to-fine event completion pipeline. First, we use an event-oriented network to generate coarse distributions of events based on conditional sparse events. Then, we use a second network to yield final completed dense events. Green and red indicate positive and negative events respectively.

### Event completion formulation

Sparsity is the intrinsic characteristic of event data, while too sparse events contain limited information for any application. The event completion task arises when event cameras capture insufficient events in challenging environments such as high-speed and dark scenarios, especially for a large-pixel-number sensor which would also encounter extreme spatial sparsity. In Eq. (1), the threshold *C* is corresponding to the reciprocal of the sensitivity *S* of the sensor. Physically raising *S* (smaller *C*) can raise the density of the sensed events but also induces more noise, which is a fundamental trade-off for event camera.

For this task, suppose ${\varvec{e}}_L$ and ${\varvec{e}}_H$ denote the events captured with $S_L$ and $S_H$ for the same scene, where $S_L<S_H$ from the latent clean dense events ${\varvec{e}}_{CD}$

2 $$\begin{aligned}&{\varvec{e}}_L={\mathscr {C}}({\varvec{e}}_{CD},S_L,\delta ), \end{aligned}$$

3 $$\begin{aligned}&{\varvec{e}}_H={\mathscr {C}}({\varvec{e}}_{CD},S_H,\delta ), \end{aligned}$$

where ${\mathscr {C}}$ is the capture process of the sensor and $\delta$ denotes the camera settings except sensitivity. As analyzed above, ${\varvec{e}}_L$ contains clean but sparse events while ${\varvec{e}}_H$ contains dense but noisy events. Both of them are imperfect for direct observation or downstream applications. The objective of enhancing event quality is to recover clean dense events ${\varvec{e}}_{CD}$ from either of these captured degraded inputs, i.e. ${\varvec{e}}_L$ or ${\varvec{e}}_H$. Event denoising task is defined as recovering ${\varvec{e}}_{CD}$ from ${\varvec{e}}_H$. Similarly, event completion task can be defined as the recovery of ${\varvec{e}}_{CD}$ from the clean sparse observation ${\varvec{e}}_L$

4 $$\begin{aligned} \hat{{\varvec{e}}}_{CD}={\mathscr {F}}({\varvec{e}}_{L},\theta ), \end{aligned}$$

where ${\mathscr {F}}$ denotes the reconstruction algorithm and $\theta$ represents its parameters. For most of the time, the paired ${\varvec{e}}_{CD}$ and ${\varvec{e}}_L$ cannot be acquired simultaneously, so we use a random sampling strategy to simulate sparse events from real dense events. Given a complete event set ${\varvec{e}}_{CD}$, the task is to reconstruct ${\varvec{e}}_{CD}$ from the down-sampled event set ${\mathscr {S}}({\varvec{e}}_{CD})$. Therefore, Eq. (4) turns into

5 $$\begin{aligned} \hat{{\varvec{e}}}_{CD}={\mathscr {F}}({\mathscr {S}}({\varvec{e}}_{CD}),\theta ). \end{aligned}$$

## Related work

### Event representation and quality enhancement

#### Event representation

Event signals have been proven to provide auxiliary help in video deblurring and frame interpolation^9–14^, image reconstruction and super-resolution^8,15,16^, and downstream applications such as object recognition^17–19^ and detection^20,21^. With the rapid development of deep learning, various event representations such as HFirst^22^, event frame^23^, event histograms^24^, event-based time surfaces^25^, event spike tensor^26^ and event volume^27^ etc have been extensively used in deep networks. Many network architectures^28–32^ that embed event streams for either image restoration or pattern recognition have also been proposed. Among these methods, temporal ordering plays an important role in the effective representation and can influence the performance of downstream applications^33–35^.

#### Event quality enhancement

Raw event signals suffer from severe noise and spatio-temporal sparsity, which challenges the visualization, analysis and downstream applications. If the camera operates in extreme cases, the quality will dramatically decrease further. To address the heavy noise, a number of methods have been proposed to denoise raw event sequences^36–42^. Other researchers attempt to super-resolve the raw events by enhancing the spatial resolution^5–8^. Considering the noise would hamper super-resolution, Duan et al.^5^ proposed a deep-learning method to jointly denoise and super-resolve neuromorphic events using an encoder–decoder network, which takes the temporally binned events as input and allocate random timestamps to the output high-resolution events. Such irreversible practice will lose temporal ordering information in the output and may harm downstream applications. As the first attempt to super-resolve events while keeping timestamps, Li et al.^6^ proposed a two-stage scheme that first acquires spatial event-count map and temporal rate function, and then obtains the event of each new pixel with a thinning based event sampling algorithm. Further, Li et al.^7^ proposed a spatio-temporal constraint learning method that optimizes the spatial and temporal event distribution based on SNN model and a simple three-layer CNN. This method achieves pleasant visual quality but requires sufficient events in the sparse input to learn the spatio-temporal distribution and is limited in millisecond resolution due to the numerical simulation of SNN^43^. Therefore, an event-to-event recovery method maintaining spatial distribution and sharp details, high temporal resolution and ordering information is highly desired.

### Point cloud completion

With the maturity of 3D sensors, point clouds have become an important form of modeling 3D scenes. A high quality point cloud is essential for downstream tasks and significant progress has been made in generating a complete point cloud from a degraded input. In the past decades, many algorithms have been proposed by using 3D CNNs^44,45^, graph CNNs^46,47^, transformer^48^. These methods learn a complete point cloud representation under direct supervision of ground truth data. In a distinct way, generative models^49–51^ etc. learn a probabilistic distribution as representation. As a new generative model, the denoising diffusion probabilistic model (DDPM)^52,53^ decomposes the generation process into multiple steps by learning to steadily denoise the random input noise. Due to its powerful generation capability, diffusion model has been applied for point cloud completion^51,54,55^ and achieved the state-of-the-art performance. As found in Ref.^55^, a conditional DDPM often generates high-quality complete point clouds that uniformly covers the shape of the target object. Inspired by DDPM’s advantageous performance and the high similarity between point cloud and event cloud, we introduce a conditional DDPM model with an event-oriented encoder-decoder network to generate a dense event sequence with fine details in a coarse-to-fine manner.

**Figure 3:**
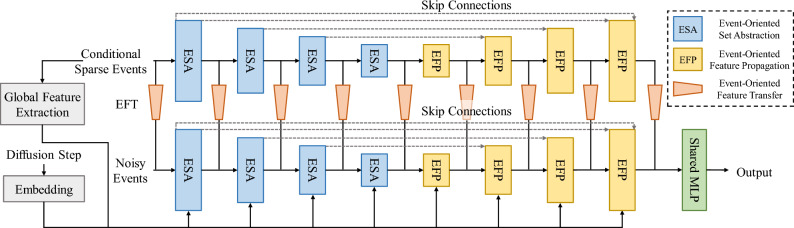
The architecture of EDR network. The upper branch extracts features from the conditional input, which is absorbed into the lower branch to denoise the noisy input. The proposed event-inspired cuboid query is extensively used in the three main modules—event-oriented set abstraction, feature propagation and feature transfer.

## Revisiting conditional DDPM

The denoising diffusion probabilistic model consists of two processes—diffusion and reverse. In the diffusion process (the blue left-arrow in Fig. 2), Gaussian noise is added to the clean complete events step by step. In the reverse process (the blue right-arrow in Fig. 2), the noise is predicted by the proposed event diffusion network and clean complete events are gradually recovered from the degraded version gradually.

### The diffusion process

Denoting the index of time steps as *j*, the Markov diffusion process from clean complete events ${\varvec{e}}_0$ to ${\varvec{e}}_J$ is defined as

6 $$\begin{aligned} q({\varvec{e}}_1,\ldots ,{\varvec{e}}_J)=q({\varvec{e}}_0)\prod _{j=1}^Jq({\varvec{e}}_j|{\varvec{e}}_{j-1}), \end{aligned}$$

where $q({\varvec{e}}_j|{\varvec{e}}_{j-1})={\mathscr {N}}({\varvec{e}}_j;\sqrt{1-\beta _j}{\varvec{e}}_{j-1},\beta _j{\varvec{I}})$, with the Gaussian noise values $\beta _j$ being pre-defined small positive constants. Following^53^, let $\alpha _j=1-\beta _j$ and ${\bar{\alpha }}_j=\prod _{i=1}^j\alpha _i$, the diffusion process $q({\varvec{e}}_j|{\varvec{e}}_0)={\mathscr {N}}({\varvec{e}}_j;\sqrt{{\bar{\alpha }}_j}{\varvec{e}}_0,(1-{\bar{\alpha }}_j){\varvec{I}})$. When *J* is large enough, ${\bar{\alpha }}_j$ approaches zero, and $q({\varvec{e}}_J|{\varvec{e}}_0)$ gets close to the latent distribution which is a Gaussian prior. Then, ${\varvec{e}}_j$ can be sampled with the simplified equation

7 $$\begin{aligned} {\varvec{e}}_j=\sqrt{{\bar{\alpha }}_j}{\varvec{e}}_0+\sqrt{1-{\bar{\alpha }}_j}{\varvec{\epsilon }}, \end{aligned}$$

where ${\varvec{\epsilon }}$ is standard Gaussian noise.

**Figure 4:**
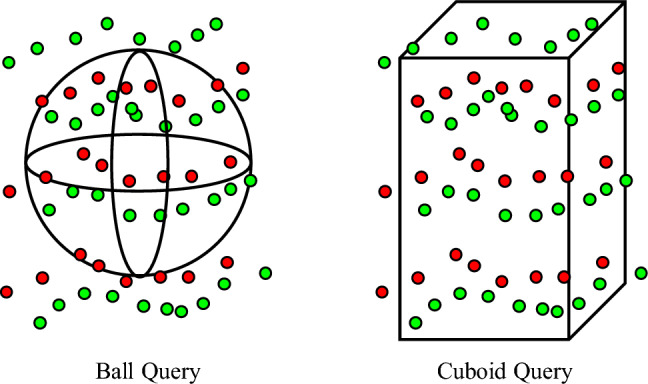
The illustration of the original ball query (left) and the proposed cuboid query (right). Cuboid query consumes more events in the temporal dimension which is important for 3D event cloud representation. Green and red indicate positive and negative events respectively.

### The reverse process

The reverse process is also a Markov process in which the added noise is predicted and removed afterwards. Conditioned on the input sparse events ${\varvec{c}}$, the reverse from noisy ${\varvec{e}}_J$ to clean events ${\varvec{e}}_0$ is defined as

8 $$\begin{aligned} p_{{\varvec{\theta }}}({\varvec{e}}_0,\cdots ,{\varvec{e}}_{J-1})=p({\varvec{e}}_J,{\varvec{c}})\prod _{j=1}^Jp_{{\varvec{\theta }}}({\varvec{e}}_{j-1}|{\varvec{e}}_j,{\varvec{c}}), \end{aligned}$$

where $p_{{\varvec{\theta }}}({\varvec{e}}_{j-1}|{\varvec{e}}_j,{\varvec{c}})={\mathscr {N}}({\varvec{e}}_{j-1};{\varvec{\mu }}_{{\varvec{\theta }}}({\varvec{e}}_j,{\varvec{c}},j),\sigma _j^2{\varvec{I}})$, with ${\varvec{\mu }}_{{\varvec{\theta }}}({\varvec{e}}_j,{\varvec{c}},j)$ and $\sigma _j^2$ denoting the predicted shape from our generative model and the variance, respectively. To generate a sample conditioned on sparse events ${\varvec{c}}$, we start from sampling ${\varvec{x}}_J$ from a Gaussian distribution and then progressively sample ${\varvec{x}}_{j-1}$ from $p_{{\varvec{\theta }}}({\varvec{x}}_{j-1}|{\varvec{x}}_j,{\varvec{c}})$ for $j=J,\ldots ,1$, and finally obtain ${\varvec{x}}_0$.

### The training process

To simplify the training objective, we follow Ho et al.^53^’s parameterization $\sigma _j^2=\frac{1-{\bar{\alpha }}_{j-1}}{1-{\bar{\alpha }}_j}$ and ${\varvec{\mu }}_{{\varvec{\theta }}}({\varvec{x}}^j,{\varvec{c}},j)=\frac{1}{\sqrt{\alpha _j}}({\varvec{x}}_j -\frac{\beta _j}{1-\sqrt{1-{\bar{\alpha }}_j}}{\varvec{\epsilon _\theta }}({\varvec{x}}_j,{\varvec{c}},j)$, in which ${\varvec{\epsilon _\theta }}$ is a neural network estimating noise from noisy point cloud ${\varvec{x}}_j$, diffusion step *j* and the conditioner ${\varvec{c}}$. The objective reduces to

9 $$\begin{aligned} {\mathscr {L}}_\text {Diff}({\varvec{\theta }})={\mathbb {E}}_{j\sim {\mathscr {U}}([J]), {\varvec{\epsilon }}\sim {\mathscr {N}}(0,1)}\Vert {\varvec{\epsilon }}-{\varvec{\epsilon _\theta }}({\varvec{e}}_j,{\varvec{c}},j)\Vert ^2, \end{aligned}$$

where ${\mathscr {U}}([J])$ is the uniform distribution over ${1,\ldots ,J}$, ${\varvec{\epsilon }}$ is the added standard Gaussian noise. The neural network ${\varvec{\epsilon _\theta }}$ can be reparameterized to predict the noise added to the clean event set ${\varvec{e}}_0$, which can be used to denoise the noisy event set: ${\varvec{e}}_j\sqrt{{\bar{\alpha }}}{\varvec{e}}_0+\sqrt{1-{\bar{\alpha }}_j}{\varvec{\epsilon }}$. During training we use ${l}_2$ loss to penalize the difference between model’s output ${\varvec{\epsilon _\theta }}({\varvec{e}}_j,{\varvec{c}},j)$ and the true noise ${\varvec{\epsilon }}$.

## Methods

In this section, we introduce the event-oriented diffusion refinement (EDR) method, a conditional denoising diffusion probabilistic model for event completion, with the overview illustrated in Fig. 2 and the key modules described in the following subsections.

### Event cloud representation

Raw event data takes the form of a sequence of four-element tuples with each event ${\varvec{e}}_r=(x, y, t, p)$, which are converted into binary points in the 3D coordinate system before being fed into the network for training or inference. Firstly, we cut the event streams sequentially into slices containing *N* events ${\varvec{e}}_r=\lbrace e_r^i; i=0,\ldots ,N\rbrace$, ranked by timestamp. Then, the event slice is normalized by the sensor’s pixel count along the spatial dimension and by the time duration along the temporal dimension, i.e.

10 $$\begin{aligned}&x^i=\bigg (\frac{x_r^i}{W-1}-0.5\bigg )\times 2, \end{aligned}$$

11 $$\begin{aligned}&y^i=\bigg (\frac{y_r^i}{H-1}-0.5\bigg )\times 2, \end{aligned}$$

12 $$\begin{aligned}&t^i=\bigg (\frac{t_r^i-t_r^0}{t_r^N-t_r^0}-0.5\bigg )\times 2, \end{aligned}$$

13 $$\begin{aligned}&p^i=(p_r^i-0.5)\times 2, \end{aligned}$$

where *W* and *H* denote the width and height of the sensor’s pixel array. So far, we can denote a sample ${\varvec{e}}$ with *N* events whose *x*, *y* and *t* values are between $- 1$ and 1. These processed events can be regarded as a set of event entries in the 3D coordinate system (similar to point cloud), as shown in Fig. 1. Besides, the polarity of each point is also attached as its feature. The network completing this set of events is built on this representation and the output conforms exactly to the same form, which can be converted back to the set of four-element tuples ordered by the timestamp.

Despite the high similarity with point cloud, the event cloud differs in multiple aspects. First of all, the *t*-dimension has different metric with spatial dimensions *x*- and *y*-, thus the event entries are unevenly distributed in the 3D space. Secondly, the normalized event points cannot form a 3D shape with smooth surfaces and have discontinuous and even scattered details instead. Besides, the events has its polarity information which is of specific meanings in physics. Therefore, we need to develop networks matching well with the unique representation.

**Table 1:** Performance comparison between our method and baseline methods.

Methods	N-MNIST		N-MNIST		Event Camera Dataset		1Mpx Detection Dataset	
	1024-256		1024-128		8192-4096		16384-4096	
	CD$\downarrow$	EMD$\downarrow$	CD$\downarrow$	EMD$\downarrow$	CD$\downarrow$	EMD$\downarrow$	CD$\downarrow$	EMD$\downarrow$
STCL^7^	14.06	17.34	18.05	18.20	55.71	27.19	13.71	24.09
PoinTr^48^	7.60	14.08	8.44	21.57	5.76	46.86	5.37	150.04
VRCNet^50^	**7.26**	16.99	**7.42**	17.28	4.33	22.39	3.49	27.90
Ablation 1	7.84	7.65	9.32	10.29	**3.16**	30.19	3.39	32.32
Ablation 2	10.59	7.10	11.66	10.66	6.14	24.13	3.74	26.93
Ours	7.99	**6.94**	9.20	**8.87**	3.58	**18.64**	**3.33**	**19.19**

CD loss indicates event-to-event difference and is multiplied by $10^3$. EMD loss penalizes distribution discrepancy and is multiplied by $10^2$.

Bold denotes the best score.

### Event diffusion-refinement network

#### The network design

Considering the high similarity between event cloud and point cloud for shape representation, we make event-oriented adaption to the point-version encoder–decoder network—PointNet++^56^ and use it as the backbone of two sub-networks, i.e. event diffusion network (EDN) and event refinement network (ERN) in Fig. 2, which complete event clouds at coarse and fine scales respectively. The detailed architecture is shown in Fig. 3. The backbone is composed of three main modules: set abstraction (SA), feature propagation (FP) and feature transfer (FT). Specifically, SA module subsamples the input event points and propagates the input features. SA block consists of a grouping layer to query neighbors for each point, a set of shared multi-layer perceptrons (MLPs) to extract features, and a reduction layer to aggregate features within the neighbors. FP module consists of a PA-Deconv module to upsample the intermediate event cloud representation, a set of shared MLPs to process features, and an attention mechanism to aggregate features. FT module transmits the information from conditional cloud to denoise the noisy input, and also consists of a grouping layer, a shared MLP and an attention mechanism to extract and aggregate features from the condition. Besides, we embed the diffusion step in the SA and FP module.

As introduced in previous sections, we pre-process a set of events (*x*, *y*, *t*, *p*) by normalizing the first three elements which fall into the range of − 1 to 1 and treating the polarity (− 1 or 1) as a feature for each event point. DDPM firstly generates 3D Gaussian noise with a random polarity feature and during model training, the noise is gradually removed and the polarity is predicted as a feature for each generated point. The main structure is the same between EDN and ERN, but the diffusion step is not used in the ERN.

To match the metric difference between spatial and temporal dimension of the event cloud, we first propose to use a cube query instead of the ball query or KNN query, encouraging the network to aggregate the events in a cube rather than a ball. In this way, the aggregated events resemble the overall distribution of all events and the network is expected to learn a better representation. Further, we lengthen the cube query along *t*-dimension, as shown in Fig. 4, to let the network pay more attention to the temporally neighboring events than those along *x*- and *y*-dimensions, because temporally adjacent events are more informative for event completion.

#### The network learning

We use the proposed EDN to generate coarse complete events and ERN for refinement. The latter predicts the relative displacement and add it to the coarse events to obtain the refined version. We use the Chamfer Distance (CD) loss between the refined event set ${\varvec{x}}$ and ground truth ${\varvec{e}}$ to supervise the learning of ERN ${\varvec{\epsilon _f}}$

14 $$\begin{aligned} {\mathscr {L}}_{\text {CD}}({\varvec{x}},{\varvec{e}})\!=\!\frac{1}{|{\varvec{x}}|}\sum _{x\in {\varvec{x}}}\min _{e\in {\varvec{e}}}\Vert x-e\Vert ^2\!+\!\frac{1}{|{\varvec{e}}|}\sum _{e\in {\varvec{e}}}\min _{x\in {\varvec{x}}}\Vert x-e\Vert ^2, \end{aligned}$$

where $|{\varvec{x}}|$ denotes the number of events in ${\varvec{x}}$. As the generation process is slow, we adopt a fast sampling algorithm^57^ to generate and save the coarse events in advance. This practice endures small performance drop compared with 1000-step generation but offers a 99.7% speedup.

**Figure 5:**
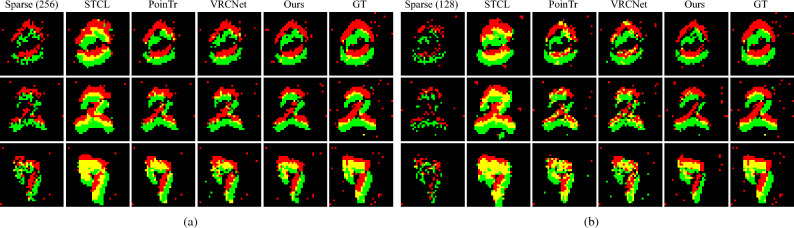
The event completion results on N-MNIST dataset from input with 256 events (**a**) and 128 events (**b**). STCL leads to too dense events which may lose local shape, e.g. ‘7’ in (**b**), while results of PoinTr and VRCNet tend to suffer from missing entries. Our method maintains both overall event completeness and local shape. Green and red indicate positive and negative events respectively.

**Figure 6:**
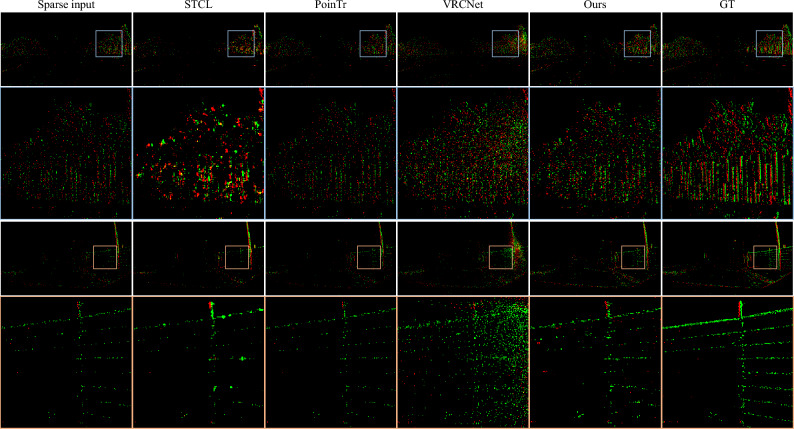
The event completion results on examples from 1 Mpx Detection Dataset. STCL leads to completed events that tend to gather around certain positions. The results of PoinTr are too sparse, while VRCNet can only learn coarse distribution. In comparison, our method can recover dense events while maintaining sharp details. Green and red indicate positive and negative events respectively.

**Figure 7:**
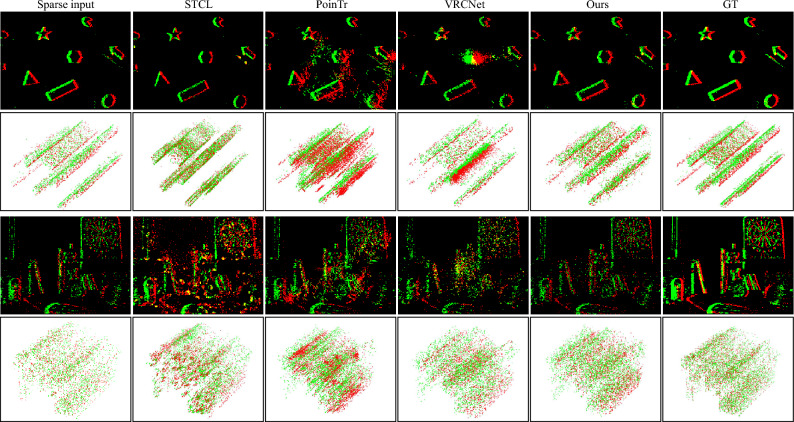
Visual illustration of event completion results and the reconstructed intensity frames on two examples from Event Camera Dataset. STCL still tends to generate unevenly distributed events gathering together, and STCL and PoinTr are inclined to generate coarse structures and lose details. Our method is free of such artifacts. Intensity frame reconstruction results also validate the superiority of our method. Green and red indicate positive and negative events respectively.

## Experiments

### Datasets

To quantitatively evaluate our method and baseline methods, we perform extensive experiments on three public event datasets, i.e. N-MNIST^58^, Event Camera Dataset^59^, 1Mpx Detection Dataset^34^ at different spatial resolutions. We also collect a dataset to test the performance in diverse real challenging scenarios.

#### N-MNIST

N-MNIST is an event version of MNIST dataset, which contains around 50,000 training samples and 10,000 test samples with 10 classes of digits, and the spatial resolution is $34 \times 34$. We use 1024 events as the ground-truth and 256/128 events as incomplete input.

#### Event camera dataset

Event Camera Dataset is composed of events captured in daily scenarios with $180 \times 240$ resolution. To avoid repetitive scenes, we select 11 snippets (50,552 samples) for training and 7 (45,388 samples) for test following^7^. Since the scenes are of complex structures and with rich semantic information, we use a 50% sampling rate to down-sample 8192-point ground-truth events to 4096 point sparse input.

#### 1 Mpx detection dataset

1 Mpx Detection Dataset is captured in a driving environment with a $720 \times 1280$ spatial resolution sensor and contains complex scenes. Since the original dataset is very large, we use 80,000 and 20,000 samples for training and test respectively. The sparse input contains 4096 points and the dense output 16,384 points.

#### Self-captured dataset

To evaluate the methods in real challenging scenarios, we capture a new dataset using an iniVation DVXplorer with resolution $480\times 640$, consisting of rich scenes including moving camera, highly dynamic objects, dim illumination etc. We include data with various challenges for training, and target to recover 16,384 events from down-sampled 4096 events during training. The training set contains 21,355 sample. We test on a continuous sequence of 4096 events to qualitatively validate the effectiveness of our approach in real scenarios.

### Baselines and metrics

Since there is no published work for event completion to the best of our knowledge, we compare our approach with a couple of closely related methods, including event super-resolution algorithm—STCL^7^ and point cloud completion algorithms—PoinTr^48^ and VRCNet^50^. STCL is originally proposed for event super-resolution and we modify its last layer to obtain output events with the same resolution as input. Besides, since STCL only has millisecond resolution, we set the simulation duration as 25 ms for 1 Mpx Detection Dataset and 50ms for other datasets. PoinTr and VRCNet are easier to be adapted for event completion. Considering that they cannot learn the polarity of event points, we assign the polarity for each entry in the completed event set according to its nearest neighbor in the input sparse events.

Since CD loss is sensitive to outliers and cannot reflect the overall distribution, we also use Earth Mover Distance (EMD) to evaluate the quality of the completed events. EMD loss penalizes the distribution discrepancy between the predicted events ${\varvec{x}}$ and the ground-truth version ${\varvec{e}}$, by optimizing a transportation problem. Specifically, it estimates a bijection $\phi :{\varvec{x}}\leftrightarrow {\varvec{e}}$ between ${\varvec{x}}$ and ${\varvec{e}}$

15 $$\begin{aligned} {\mathscr {L}}_{\text {EMD}}({\varvec{x}},{\varvec{e}})=\min _{\phi :{\varvec{x}}\leftrightarrow {\varvec{e}}}\sum _{x\in {\varvec{x}}}\Vert x-\phi (x)\Vert _2. \end{aligned}$$

Comparatively, EMD is more appropriate for measuring the distance between two distributions.

Despite the fact that CD and EMD are originally for measuring point cloud distances and currently, there is no perfect metric for event sequence as far as we know, both of them are able to measure the distance between 3D event data (*x*, *y*, *t*) since raw 4-element tuple are already converted to 3D event points with 1/− 1 polarity feature after normalization. By definition, CD loss does not require two event sets to contain the same number of events and can measure data with any dimension number. Therefore, the use of CD for training is feasible in our method. As a supplement, we use EMD to penalize the overall distribution for 3D event points.

**Figure 8:**
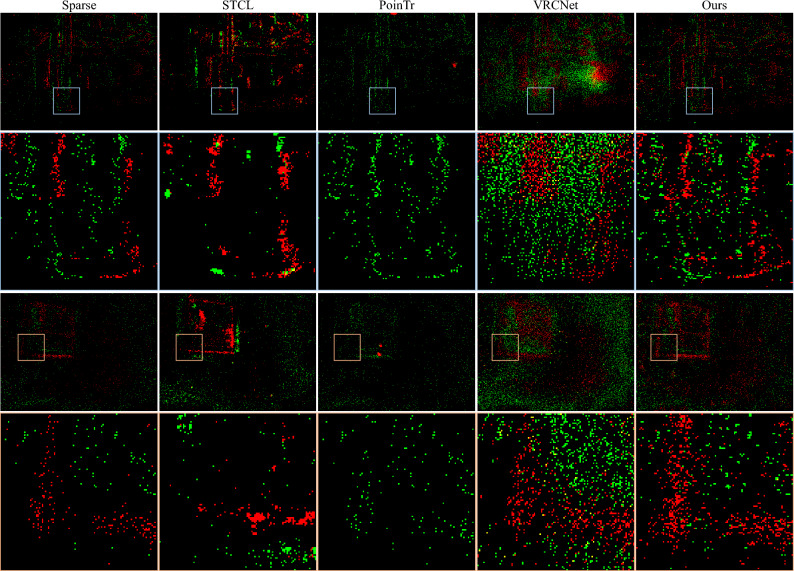
Visualization of completed events on two examples from the self-captured dataset. Top: a tea table captured by a DVS on a rotating stage at $360^\circ$/s. Bottom: a painting placed in a dark room. Green and red indicate positive and negative events respectively.

### Implementation details

We learn our model in a coarse-to-fine manner. Firstly, we train the coarse network for 120 epochs for other three public datasets and 300 epochs for the self-captured one, with learning rate of $2e^{-4}$ using Adam optimizer. Since generating a sample with 1000 steps is too time-consuming, we adopt a fast sampling method—DPMSolver^57^ for acceleration and generate a sample after only 27 steps with slight performance degradation. Afterwards, we feed the generated coarse event clouds into the refinement network, which takes 30 epochs to converge. We empirically found the optimal *t* of the proposed cuboid query varies for different datasets. Let the bottom edge length be *r*, the optimal length of *t* dimension is 1*r*, 1*r*, 1.2*r* and 1.5*r* for the four datasets respectively.

### Event completion results

#### Quantitative results

In Table 1, we report the CD and EMD loss of our method and baseline algorithms. Specifically for datasets, on the N-MNIST dataset, STCL leads to obviously higher scores of both CD and EMD metrics. PoinTr and VRCNet obtain lower CD losses while higher EMD losses than the proposed method. The reason may be that the event sequences in N-MNIST are in low spatial resolution (only $34 \times 34$ pixels), and distributed more evenly in the temporal dimension than the other two complex high-resolution datasets. The resemblance between such data and conventional point cloud data leads to good performance for CD loss. Still, our proposed method can learn a better distribution according to EMD loss. On the Event Camera Dataset, STCL leads to an order higher CD loss than the other methods, indicating the completed events are coarse. Compared to the point-based PoinTr and VRCNet, the proposed method achieves lower CD loss and EMD loss. On the 1 Mpx Detection Dataset, our proposed method still produces the highest CD and EMD scores among all the algorithms.

STCL leads to higher CD and EMD compared to the point-based method. It is attributed to the fact that STCL is limited to millisecond resolution in SNN simulation, so cannot learn the latent structure of the events sparsely distributed in both spatial and temporal dimensions. Instead, it is more appropriate for recovering high-resolution event points from dense data. As modern point completion networks, PoinTr and VRCNet yield low CD losses on the three datasets, since they use CD loss for optimization. However, the EMD losses are very high, which indicates that it encounters difficulty in completing complex event data with high spatial resolution and sharp details. Still, we notice that our event-oriented method achieves the best EMD across all groups of experiments for all datasets and comparative CD loss to the second competitor, which validates the feasibility of the generative model in predicting missing events and demonstrates that event-specific modules can better represent the distribution of event data. In sum, the superiority of our method is attributed to both the generative nature and event-oriented modules.

#### Qualitative results

We visualize the completed event data by accumulating the completed events into 2D frames, as shown in Figs. 5, 6, 7 and 8. STCL leads to pleasant results for most cases, but it tends to generate occluded structures. In many situations, the predicted events densely gather at certain locations, losing the sharp thin structures. Although PoinTr and VRCNet obtain low CD loss, the visual results are unpleasant. The visualization indicates that PoinTr fails to learn the data shape or distribution for the whole event set, and instead, the adopted point-wise loss misleads the completed points to adhere to the input sparse events. VRCNet learns coarse distribution but fails to recover sharp details. In comparison, our proposed model can recover details and sharp edges for all datasets. Especially on the self-captured dataset, as shown in Fig. 8, baseline methods fail to complete informative and sharp shapes, but our method achieves promising visual results in challenging high-speed and low-illumination environments. For example, the legs of the tea table (captured by a DVS on a high-speed rotating stage), the contents and the frame of the painting (captured in a dim room) are all clearly reconstructed. Based on the platform for data collection, our method supports the completion of event data captured at a rotation speed of $360^\circ$/s and with an illuminance of 3 lux.

### Ablation study

Firstly, we conduct an ablation study by replacing the event-oriented cuboid query with a ball query. The EMD loss rises by 0.71, 1.42, 11.55, 11.16 on N-MNIST (256), N-MNIST (128), Event Camera Dataset, and 1Mpx Detection Dataset, respectively although CD loss is similar, as shown in Table 1 indicated as Ablation 1. On the N-MNIST dataset, ball query leads to slightly lower CD loss but also higher EMD loss compared to the proposed cuboid query. The event sequences in N-MNIST are in low spatial resolution and distributed more evenly in the temporal dimension. These data are very much like conventional point clouds, usually 3D shapes with smooth surfaces. In such case, ball query can better represent and encode such easy evenly distributed data. However, cuboid query is more suitable for real challenging event datasets with complex scenes. It is also flexible for various datasets, since the t-dimension length of the cuboid query can be adjusted according to data. The results quantitatively validate the contribution of event-oriented cuboid query for effective event representation.

Secondly, to validate the importance of refinement network, we conduct an ablation study by removing the ERN and comparing the results. We summarize the results in Table 1, indicated as Ablation 2. The results tell removing the ERN leads to higher CD and EMD losses on all three datasets compared to the full EDR model, which demonstrates the necessity of the refining process.

### Downstream applications

To further demonstrate the benefits brought by our event completion method with precise timestamps for the downstream tasks, we conduct two downstream experiments—object classification and intensity frame reconstruction, on the completed event streams.

#### Object classification

We test the completed results of N-MNIST for digit classification. We train an object classification network for event data^26^ using complete dense events, and test on the generated events by all methods. The results are shown in Table 2. The 1024-event dense cloud achieves 99.1% accuracy on the test set. For the 256-event setting, three methods achieve over 90% accuracy except for VRCNet. STCL obtains 96.7% accuracy, while our model leads to 96.0% accuracy. When the number of input points decreases to 128, the accuracy of PoinTr declines dramatically, while the other two still result in over 90% accuracy. The coarse prediction of both shape and polarity induces low classification accuracy in the 128-point case. VRCNet leads to the lowest accuracy in both settings. In comparison, our method can regress the polarity of each generated event point and high-fidelity shape, which assures the accuracy of this task. Compared to STCL, our method is more robust to the sparsity of input events.

#### Intensity frame reconstruction

We reconstruct intensity frames from the completed results of Event Camera Dataset using E2VID^3^ and report the PSNR and SSIM^60^ of the results from completed events compared with the reference from ground-truth events in Table 3. Our method achieves the best PSNR for most of the settings and the best SSIM in all settings.

**Table 2:** Comparison of classification accuracy on the N-MNIST dataset, using the model trained on high-resolution events to test the completed clouds and LR input.

Methods	N-MNIST	
	1024-256 (%)	1024-128 (%)
LR	53.6	28.6
STCL	**96.7**	90.8
PoinTr	92.8	59.4
VRCNet	76.2	45.0
Ours	96.0	**91.1**
GT	99.1	

Bold denotes the best score.

**Table 3:** Comparison of reconstructions on the event camera dataset in terms of PSNR and SSIM.

Scenes	PSNR$\uparrow$				SSIM$\uparrow$			
	STCL	PoinTr	VRCNet	Ours	STCL	PoinTr	VRCNet	Ours
boxes_6dof	20.50	8.91	21.39	**21.69**	0.33	**0.46**	0.36	**0.46**
calibration	17.70	14.97	17.37	**18.24**	0.41	0.50	0.46	**0.58**
dynamic_6dof	16.68	16.17	17.66	**18.87**	0.47	0.67	0.45	**0.70**
office_zigzag	**20.68**	15.67	16.29	17.56	0.56	0.59	0.45	**0.61**
poster_6dof	19.47	8.81	19.36	**19.87**	0.37	0.52	0.39	**0.54**
shapes_6dof	**21.11**	18.57	19.58	17.42	0.80	0.82	0.83	**0.88**
slider_depth	**22.46**	15.30	16.15	21.11	0.69	0.67	0.55	**0.74**
Average	19.42	10.96	19.65	**20.23**	0.40	0.54	0.41	**0.55**

Bold denotes the best score.

## Conclusion

In this paper, we target for addressing the lacking density of event streams in challenging cases (e.g., high-speed and low-light conditions) by introducing an event-oriented diffusion-refinement method for event completion to rebuild the missing events. We formulate an event stream as a 3D cloud and design an event-oriented conditional diffusion probabilistic model to generate the completed event points in a coarse-to-fine manner. To the best of our knowledge, this is the first work defining and exploring this task. We compare our method with relevant algorithms to validate its superiority both quantitatively and visually. Furthermore, the performance on two downstream applications, i.e. object classification and intensity frame reconstruction, demonstrates the usability of our method. Our approach would unlock the potential of event cameras and broaden their applications.

Due to the multi-step sampling process during inference, the generation of coarse events is rather slow, so the training/inference of the proposed method cannot be realized on the fly. In the future, faster and better sampling mechanism can be applied to enable end-to-end training/inference, which will further permit real-time event completion such as on-board deployment.
